# Front-of-pack (FOP) labelling systems to improve the quality of nutrition information to prevent obesity: NutrInform Battery vs Nutri-Score

**DOI:** 10.1007/s40519-021-01316-z

**Published:** 2021-10-19

**Authors:** Michele O. Carruba, Antonio Caretto, Antonino De Lorenzo, Giuseppe Fatati, Andrea Ghiselli, Lucio Lucchin, Claudio Maffeis, Alexis Malavazos, Giuseppe Malfi, Enrica Riva, Chiara Ruocco, Ferruccio Santini, Marco Silano, Alessandra Valerio, Andrea Vania, Enzo Nisoli

**Affiliations:** 1 Center for Study and Research on Obesity, Department of Biomedical Technology and Translational Medicine, University of Milan, Milan, Italy; 2 Endocrinology, Metabolic Diseases and Clinical Nutrition, Hospital of Brindisi, Brindisi, Italy; 3 Division of Clinical Nutrition and Nutrigenomic, Department of Biomedicine and Prevention, University of Tor Vergata, Rome, Italy; 4 Italian Obesity Network, Orvieto, Italy; 5 Society of Alimentary Sciences, Rome, Italy; 6 Regional General Hospital, Bolzano, Italy; 7 Department of Surgery, Dentistry, Paediatrics and Gynecology, University and Azienda Ospedaliera Universitaria Integrata of Verona, Verona, Italy; 8 Endocrinology Unit, Clinical Nutrition and Cardiovascular Prevention Service, IRCCS Policlinico San Donato, San Donato Milanese, Milan, Italy; 9 Department of Biomedical, Surgical and Dental Sciences, University of Milan, Milan, Italy; 10 Department of Dietetics and Clinical Nutrition, San Giovanni Battista Hospital, Turin, Italy; 11 Italian Society of Paediatric Nutrition, Milan, Italy; 12 Obesity and Lipodystrophy Center, Endocrinology Unit, University Hospital of Pisa, Pisa, Italy; 13 Unità Operativa Alimentazione, Nutrizione e Salute, Dipartimento Sicurezza Alimentare, Nutrizione e Sanità Pubblica Veterinaria, Istituto Superiore di Sanità, Rome, Italy; 14 Department of Molecular and Translational Medicine, Brescia University, Brescia, Italy; 15 Department of Paediatrics and Paediatric Neuropsychiatry, La Sapienza” University of Rome, Rome, Italy

**Keywords:** Dietary choice, Education, Food, Food portions, Nutrition, Obesity, Participation, Policy, Prevention, Public health

## Abstract

**Abstract:**

Many systems for classifying food products to adequately predict lower all-cause morbidity and mortality have been proposed as front-of-pack (FOP) nutritional labels. Although the efforts and advances that these systems represent for public health must be appreciated, as scientists involved in nutrition research and belonging to diverse Italian nutrition scientific societies, we would like to draw stakeholders’ attention to the fact that some FOP labels risk being not correctly informative to consumers’ awareness of nutritional food quality. The European Commission has explicitly called for such a nutrition information system to be part of the European “strategy on nutrition, overweight and obesity-related issues” to “facilitate consumer understanding of the contribution or importance of the food to the energy and nutrient content of a diet”. Some European countries have adopted the popular French proposal Nutri-Score. However, many critical limits and inadequacies have been identified in this system. As an alternative, we endorse a new enriched informative label—the NutrInform Battery—promoted by the Italian Ministry of Health and deeply studied by the Center for Study and Research on Obesity, Milan University. Therefore, the present position paper limits comparing these two FOP nutritional labels, focusing on the evidence suggesting that the NutrInform Battery can help consumers better than the Nutri-Score system to understand nutritional information, potentially improving dietary choices.

**Level of evidence:**

II. Evidence was obtained from well-designed controlled trials without randomization.

## Introduction

Obesity and its health consequences are highly prevalent in Europe. Such conditions are unevenly distributed among the population, being more frequent in the less favourite socio-economic and less educated classes [1, 2]. It is well known that excess body weight is a multi-factor disease. Within an overall approach that considers the entire lifestyle, dietary control is probably the most helpful tool to prevent this condition, which is complex to handle clinically due to the limited availability of effective therapeutic approaches [3]. Interventions to improve the population’s knowledge of proper nutrition and lifestyle are necessary to reduce the risk of developing obesity and its consequences [4].

Among the possible informational/educational interventions, supplementary nutrition labelling systems to be printed on the front of packages of food products (FOP: front-of-pack) have been elaborated and confronted in the last few years, including Reference Intakes (Reference intakes implemented by certain manufacturers in different countries since 2006), Warnings (Health warning symbol implemented in Chile since 2016), Nutri-Score (adopted in France since 2017, and then in Belgium, Spain, Germany, the Netherlands, Luxembourg, and Switzerland between 2018 and 2020), Health Star Rating (HSR, System of classification of health stars: implemented in Australia and New Zealand since 2014), and Multiple Traffic Light (MTL, Multiple Traffic Lights implemented in the UK since 2005) [5–11]. Similarly, the European Commission (EC) has explicitly called for such a nutrition information system to be part of the European “strategy on nutrition, overweight and obesity-related issues” to “facilitate consumer understanding of the contribution or importance of the food to the energy and nutrient content of a diet” thus allowing consumers to operate healthier food choices as requested by the EC (Regulation 1169/2011).

A discussion is underway within the EC to identify and choose the adequate FOP system to reach such a goal (https://ec.europa.eu/food/safety/labelling-and-nutrition/food-information-consumers-legislation/nutrition-labelling_en), by considering that the goal of FOPs is much broader than that. Proper nutrition—as interpreted by FOPs—reduces the incidence of non-communicable diseases (NCD), such as cardiovascular diseases, cancer, chronic respiratory diseases, and diabetes, that represent major causes of disability, ill-health, health-related retirement, and premature death in the European Union (EU), resulting in considerable social and economic costs (https://ec.europa.eu/health/non_communicable_diseases/overview_en). This debate is quite relevant for the population since the chosen FOP system will become mandatory throughout the EU, thus influencing people’s food purchasing behaviours and the overall quality of the information on nutritional characteristics of food. The debate focuses on the choice between systems with different characteristics, i.e. interpretative or informational.

In this context, the Center for Study and Research on Obesity (CSRO) at the University of Milan (Italy) − whose primary mission is to promote scientific research, correct nutritional information, and political advice on obesity and related disorders [12, 13] − deemed it necessary to provide a strictly scientific analysis of the FOP systems to allow “consumers to operate healthier food choices”, as requested by the EC. Consequently, CSRO has elaborated a document describing a new enriched informative label—the NutrInform Battery—alternative to other nutrition labelling systems. Many Italian scientific societies of nutrition and metabolism have endorsed or approved in general this document to provide the Italian Health Ministry and institutions with scientific, evidence-based opinion to discuss FOP systems functional to help to combat the obesity epidemics in Europe (see the list of the Italian scientific societies supporting the present document).^1^

## The debate on FOP to be adopted to improve weight control in the European population

The ongoing debate is essentially focused on choosing between a supplementary nutritional labelling system of interpretative (or evaluation) type, Nutri-Score and an informational system proposed by our country, NutrInform Battery. Thus, we have summarised the recently published evidence on the efficacy of these two FOP systems in favouring the comprehension of nutrition relevance on health, and particularly on obesity in the consumers.

**Fig. 1:**
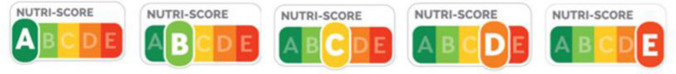
The Nutri-Score labelling system. This system is a summary, colour-coded, graded FOP label that shows a scale of five colours, from dark green to red. The Nutri-Scoresystem combines positive characteristics (i.e., fruit, vegetables and nuts, fibre, protein and seed, walnut and olive oils content) with negative characteristics (i.e., energy, total sugar, saturated fatty acids and sodium content) to achieve a score between – 15 (most healthy) and + 40 (least healthy). As shown, this score is reduced to a combination of a letter (A to E), where A reflects the highest nutritional quality and E the lowest

Nutri-Score is a supplementary nutrition labelling system of foods, developed by French researchers and adopted by France, based on a synthetic indicator: a letter from A to E, matched to colour, from green to red, which summarises the proposed nutritional profile of 100 g of each food product [14]. With a specific algorithm, Nutri-Score assigns negative points to products based on their content in calories, sodium, sugar and saturated fats (per 100 g) and positive points based on their content of fibres, proteins and selected ingredients (fruit, vegetables, legumes, nuts and seeds, olive and walnut oils) [15]. Based on the obtained final score, each food is classified into five categories: from the best (A, dark green) to the worst (E, red) (Fig. 1).

According to promoters, Nutri-Score conveys a simple message of easy comprehension to the public. However, several criticisms of this system can be raised. First of all, Nutri-Score is highly focussed on the content of nutrients with “unfavourable” effects, which confer up to 40 negative points, impacting the final score much larger than nutrients with “favourable” effects, which bear a maximum of 15 positive points. Consequently, a system primarily focuses on what persons should not eat rather than what they should eat. Thus, the NutriScore system is clearly in contrast with the most recent scientific data, such as those published on Lancet by an authoritative international research group, the Global Burden of Diseases (GBD), stating “*This finding suggests that dietary policies focusing on promoting the intake of components of diet for which current intake is less than the optimal level might have a greater effect than policies only targeting sugar and fat, highlighting the need for a comprehensive food system interventions to promote the production, distribution, and consumption of these foods across nations*” [16].

GBD data suggest that among the 15 nutritional factors with the most significant impact on the health of populations (death risk included), ten refer to foods and nutrients consumed in insufficient quantity, and only 5 to foods consumed in excessive quantity; except for sodium (i.e., salt), moreover, these foods/nutrients have a minimal (and almost irrelevant) weight on the health in countries such as Italy (about 0.86% of food-attributable deaths, according to GBD) [16]. Notably, according to Nutri-Score, saturated fats, which contribute significantly to the negative points, are not present in the list of nutrients presented by GBD to be reduced to improve health. According to a study published a few years ago, this situation also characterises Italians’ most frequent food mistakes, who are deviating more frequently from the norms dictated by the Mediterranean Diet [17]. Therefore, the “positive points” should weigh much more than the “negative points” evaluating foods: precisely the opposite that occurs when using Nutri- Score.

Moreover, the decision of Nutri-Score to evaluate 100 g of a product instead of a food serving (which is, on the opposite, at the centre of the NutrInform Battery system proposed by the Italian government) needs to be carefully considered. The role of food portion size concerning overweight and obesity in children and adults has been widely investigated and recognized [18, 19]. Despite this knowledge, Nutri-Score assigns a final “judgement” (letter or colour) based on the composition and the characteristics of a quantity of food (i.e., 100 g) that, in most cases, does not correspond to the actual portion, that can be much higher or much lower. As a consequence, there are foods (i.e., vegetable pizza) that obtain a favourable score for 100 g but are generally consumed in much higher quantities and can have a more significant impact on the overall quality of the diet in terms of calories and nutrients, compared to other foods (i.e. chocolate) that obtain (always on 100 g) less favourable scores using the Nutri-Score system but are generally consumed in much smaller portions. This critical point is also highly evident for olive oil consumption [see 20]. Even if olive oil is highly caloric, and as Nutri-Score’s algorithm appraises its content of 100 g, the average consumption of olive oil is no more than 40–45 g per day, biasing the results. Moreover, Nutri-Score does not differentiate between refined and extra virgin olive oil, seed oil, and walnut oil. This lack of distinction also opposes the well-known properties of extra virgin olive oil [21, 22].

Another critical point is that compared to what could have been anticipated based on the results of laboratory studies and in hypothetical settings, the effects of the Nutri-Score label were minor in a large-scale randomised controlled trial examining whether four pre-selected FOP nutrition labels—including SENS (Système d'Etiquetage Nutritionnel Simplifié [simplified nutrition labelling system]), Nutri Repère, Nutri-Couleurs beyond Nutri-Score—improve food purchases in real-life grocery shopping settings [23]. As reported by the authors, using the food standard agency (FSA) nutrient profiling score, the Nutri-Score system improved 0.142 FSA points, a 2.5% improvement of the average FSA score of 5.61. Moreover, the Nutri-Score's effects, like those of the other three labels, were made principally by the freshly prepared food category, a category with the widest variance in the nutrition quality [23].

Finally, Nutri-Score is based on the “a priori” classification of nutrients and foods as favourable and unfavourable. This concept contrasts with the opinion, now widely accepted in the scientific community, that diet should be evaluated in its complexity (see, for example, the Mediterranean diet) rather than examining the single foods that are part of it. Indeed, although there are foods with more or more minor optimal nutritional characteristics, the best approach to prevent or control overweight/obesity—as well as the NCD—is likely based on the conscious selection of foods and (or especially) on their rational combination in appropriate amounts and frequencies consumption. These concepts have been recently reviewed in a rigorous commentary article, listing several pitfalls and oversimplifications of the current approaches to nutrient profiling and the dichotomic classification of foods into "healthy" and "unhealthy" products [24].

## Which information does Nutri-Score genuinely provide to the consumer?

Nutri-Score is a merely interpretative and non-educational/informative system: it does not improve the consumer’s knowledge or nutritional information because the principles of the scoring system are not known (and would be unintelligible in any way) to the consumer and must hence be accepted in a non-critical way. Furthermore, it does not provide any assistance in deciding the overall diet composition, nor does it facilitate in any way an appropriate combination of various foods.

Moreover, Nutri-Score does not identify the component primarily responsible for the product’s final rating, either favourable or unfavourable. Therefore, it does not provide helpful indications (differently from NutrInform Battery) to people or patients with specific nutritional needs. Individuals with overweight or obesity will not find specific information regarding the energy content of foods (and specifically per portion calories). Similarly, subjects with high cholesterol levels will not be able to assess the content of saturated fats in their diet, or the individuals suffering from hypertension will not be able to evaluate the content of sodium in foods, or people with diabetes will not be able to find information about the amount of simple sugar in meals, except after a systematic search and comprehension of the nutritional label if it is present. Such a situation will make it very complex (or even impossible) for individuals to identify foods more or less suitable for their specific needs. Not surprisingly, the published evidence reporting positive effects of adopting the Nutri-Score system, stating that it can favourably impact health endpoints at a population level (specifically to prevent overweight and obesity), is relatively weak. For example, in the study by Julia et al. [25] based on SU.VI.MAX cohort, the subjects with the best dietary pattern according to Nutri-Score, compared to those with the worst dietary pattern, boast a lower energy intake at the beginning of the observation period but a higher prevalence of overweight and obesity, with slight and poorly defined differences in physical activity. The data are difficult to explain and can only be understood by assuming a systematic underestimation of energy intake in the group with a better diet, or an overestimation in the group with a worse diet, or a critical reverse-causality phenomenon: all conditions that limit the validity of the data collected. The authors focus their practical observation on body mass index (BMI) evolution during a 13-year follow-up period, observing an increase of this parameter over 0.62 points (0.70 after a complete statistic adjustment) in the quartile with the better pattern. However, raw data are not provided, from which it would stand out that the absolute difference (after 13 years) between the first and fourth quartile is irrelevant (+ 0.10 points) since, in practice, only the BMI difference between the two groups at the beginning of the study was annulled, when the quartile with worst diet pattern had, as reminded, a BMI lower than 0.52 points compared to the quartile with the better pattern. It is also interesting to notice that the second quartile, characterised by the worst diet pattern compared to the first, shows BMI values 0.29 points lower (hence better), always compared to the first quartile (24.03 vs 24.32), with a difference that grows further during the years, dropping off an additional 0.31 points (or 0.21 after a complete statistic adjustment) and bringing the difference to -0.60 points (or -0.50 after a complete statistic adjustment). The subjects of the second quartile, with a diet pattern worse by definition than the first one, had a better BMI at the beginning of the studies, which further improved during the 13-year follow-up, always compared to the subjects of the first quartile. This fact introduces a J trend in the relation between the diet quality measured through Nutri-Score and weight trend, which stands out to be similar in the studies in which the effect on mortality for all causes is examined. This result is difficult to understand.

The scenario that stands out from the NutriNet-Santé cohort is different. It was examined in Egnell et al. [26], in which the tertile with worse diet quality has a higher energy intake (about + 15%) than the tertile with better diet quality, a BMI slightly higher and a slightly higher prevalence of obesity. Over time, this tertile with worse diet quality shows an increase of BMI of about 0.5 points higher than the value observed among subjects with better diet quality. Since the energy intake is higher in the third group than the first group, this higher weight increase can also be explained based on the differences in the energy intake.

The relation between BMI, energy intake and healthiness index of the diet observed in Julia et al. study is similar to what has been observed in other studies conducted on SU.VI.MAX cohort [27, 28]. In this study, in particular, the subjects of the quintile with better diet pattern according to Nutri-Score, compared with the quintile with worst diet, are characterised, at the beginning of the observation period (when they are in average 50.8 vs 47.5 years old) by a lower energy intake (1,767 vs 2,112 kcal without considering alcohol, and 1,865 vs and 2,277 kcal also considering alcohol) but higher prevalence of overweight (33.0 vs 25.8%) and obesity (7.7 vs 5.2%), in the presence of slight differences in the physical activity. These results mean that the group classified with a healthier diet has underreported its food consumption at least in quantitative terms compared to the group with a less healthy diet; therefore, their report will likely be inaccurate in qualitative terms. These selective differences in reporting the diet in the various groups make the various clinical endpoints' variations unreliable.

Therefore, one can conclude that the works, based on SU.VI.MAX study, do not allow to document any favourable effect in adopting Nutri-Score on BMI at the beginning of the study (instead, BMI decreases with the reduction of the quality level of the diet); the effects observed in the longitudinal follow-up of the study are irrelevant (only annul the negative differences detected at the baseline) and are characterised by a non-linear trend that is difficult to understand.

## The Italian proposal

**Fig. 2:**
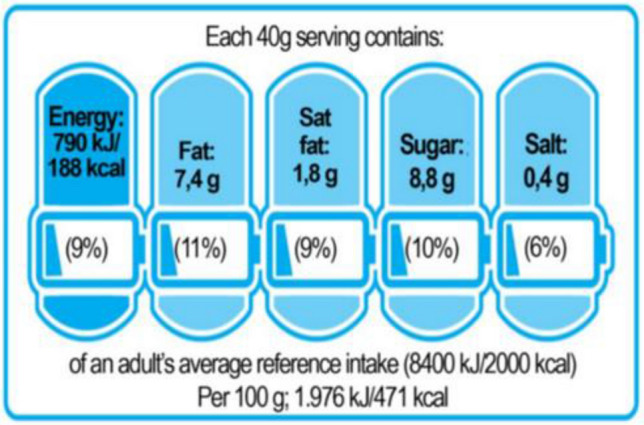
The NutrInform Battery labelling system. To be underlined that all values expressed are relative to the individual serving of 40 g. Each box contains a quantitative indication of the individual portion's energy, fat, saturated fat, sugar, and salt content. The energy content is expressed both in joules and calories. The contents of fat, saturated fat, sugar and salt are expressed in grams. The " battery" symbol shows the percentage of the individual portion's energy, fat, saturated fat, sugar and salt toward the recommended daily intake. The recommended daily intake amounts in the EU are energy, 8400 kJ / 2000 kcal; fat, 70 g; saturated fat, 20 g; sugars, 90 g; salt, 6 g. The charged portion of the battery graphically represents the percentage of energy or nutrients contained in the individual portion, allowing you to quantify it visually as well. The sum of what one eats during the day can "fill" the battery charge without going overboard, not to exceed the recommended daily intake

The project on NutrInform Battery, the FOP system proposed by Italy—led by four Italian ministries (Health, Economic Development, Agriculture, and Foreign Affairs), and opposed to Nutri-Score system—has been carried out by nutritional experts from the Istituto Superiore di Sanità and the Consiglio per la Ricerca Economica e Alimentare; in addition, representatives of trade associations from the agri-food industry and consumers have been involved (https://www.nutrinformbattery.it/). This labelling system is, contrary to Nutri-Score, an informational (non-directive) system based on the description of the food serving consumed. NutrInform Battery displays, through a simple system of five pictograms with the shape of a battery: total calories, total fats, saturated fats, sugar and salt contained in a standard portion of the considered food, as both absolute amount and percentage of the total daily intake, indicating for each food product the “filling” level of the five batteries (Fig. 2). The reference parameters on which the individual batteries are calibrated (one for each critical element, i.e. calories, total fat, saturated fat, sugars and salt) are the European ones set in Table XIII of EU Regulation no. 1169/2011—art. 35, which are also in line with LARN's (reference levels of nutrients for the Italian population). The portions determined based on available scientific nutritional evidence are derived. The filling level corresponds, in fact, to the percentage of that specific nutrient that the recommended portion of the food brings to the consumer's diet, referring to the Reference Intakes (part B of Annex XIII of EU Regulation 1169/2011). Therefore, by simply looking at each battery, the consumers can immediately see the exact percentage of the nutrient they are taking in with the portion of food consumed compared to the maximum recommended amount, and therefore how much of that nutrient they have left to consume during the rest of the day. Consequently, NutrInform Battery focuses the consumer’s attention on the nutrient content of the serving of eaten food, without prohibiting or promoting any of them in particular, but providing information on how and to what extent that food serving will affect his/her daily food intake. The aim is to guide the consumers towards more informed nutritional choices and help him/her to improve their dietary knowledge, in agreement (and not in contrast) with the strategies that public health agencies and scientific societies of various countries are implementing to promote nutrition education.

In our opinion, the merit of NutrInform Battery is also its capacity to allow and promote the proper combination of various foods (e.g., the choice of food for which the system assigns a high content of fats and sugar can be “balanced” by eating other foods with lower content of these nutrients) and to select foods, when relevant, according to specific individual needs (energy content, sodium, or saturated fat content). These actions – as already stated – cannot be made by adopting the Nutri-Score labelling system.

Therefore, the NutrInform Battery label helps the adult and responsible consumer to be aware of the food servings and encourages the food industry not only to reformulate potentially health-critical products but also to reduce the portion sizes, which may eventually orient consumers to prefer foods with lower impact on their daily calorie intake. The scientific literature, in fact, clearly shows that the generalised increase in portion sizes is responsible for excessive food consumption. On the other hand, reducing the size of the servings could effectively reduce the amount of eaten food [18]. This consideration seems particularly relevant in the school food environment. Policy actions, including adequate FOP labelling systems for the snacks found in vending machines, are needed to improve children's eating behaviour and BMI [29, 30].

The Italian FOP labelling system has been tested among consumers from seven European countries for comprehension, satisfaction, and ability to address better choices; the results of these studies confirm that this is a comprehensible and straightforward system that allows making healthier choices [31]. NutrInform Battery proved to help consumers understand and retain information, obtaining better performances in all countries where it was tested compared to Nutri-Score. It turned out to be more informative and valuable [32].

An additional piece of information needs to be discussed. Labelling systems based on colours, such as Nutri-Score, are well seen by consumers and many stakeholders. According to the authors' intentions, these labels should help the consumer operate healthier choices during purchase. Evidence in the literature shows that consumers welcome this system positively, increasing the purchase of foods labelled in green and decreasing foods marked in red [33, 34].

Today, several studies on the effect of food labels clearly show how the consumer associates the green light to the meaning of “healthy”, “natural”, “light”, thanks to the positive vibe linked to the green colour and how this association can influence opinions on health, regardless of the nutritional information indicated on the label. Nonetheless, it must be reminded that this behaviour is not necessarily positive for the consumers and could theoretically expose them paradoxically to a higher risk. Research has also demonstrated that when the packaging for the same product is experimentally prepared with two different labels, one green and one red, consumers choose the product labelled in green and does not read the information featured on the nutritional label [35].

This behaviour has already been described for other food products, such as the so-called “light foods”, whose association with alleged healthier qualities could contribute to developing obesity rather than prevent it [35–38]. Hence, these products are perceived as healthier, and the consumed amount of these foods might be more extensive. On the contrary, the FOP labelling system proposed by Italy, NutrInform Battery, focuses the consumer’s attention on the proper amount of the given food to be consumed, providing the necessary information to understand how that serving will affect the total daily intake. Hence, correct information cannot simply classify foods as good or bad (as it happens with Nutri-Score, at least as it has been proven to be read by the consumer) but should educate to balance qualitative and quantitative aspects, considering portions and consumption frequencies.

Some possible limitations of this labelling system may be suggested and are to mention. Visually, the graphics of the NutrInform Battery system could be challenging to read due to the numerous numerical references present; additionally, it may request basic nutritional knowledge. Furthermore, such a labelling system evaluates the single portion (the weight of which can sometimes vary from one manufacturer to another). Thus, it would only allow a correct comparison between categories of similar products in identical quantities. Finally, it should be remembered that the Italian decree (19.11.2020, in the form of presentation and conditions of use of the NutrInform Battery labelling system) provides for specific exclusions, for example, foodstuffs packaged in packages whose largest surface area is less than 25 cm^2^, products covered by Regulation (EU) No 1151/2012 (PDO, Protected Designation of Origin; PGI, Protected Geographical Indication, and TSG, Traditional Speciality Guaranteed), to prevent consumers from not understanding or recognising the quality mark due to the affixing of an additional logo. These issues need to be addressed and resolved to serve the consumers of every European country better.

## Conclusion

In conclusion, given this overall body of evidence, we deem it extremely important that the scientific communities, in particular nutrition scientists and experts devoted to obesity, intervene in the debate ongoing at the EC level to stress how, based on the evidence available, the FOP labelling system proposed by the Italian government and called NutrInform Battery, should be considered the most appropriate tool to increase the consumers’ nutritional knowledge and to provide them with the information necessary to plan probably with better results, the prevention of overweight and obesity. The public health community and policy-makers have repeatedly stated the importance of implementing policies based on research evidence. As reported here, multiple studies have been conducted, showing strengths and limitations for both Nutri-Score and NutInform Battery systems. However, the NutrInform Battery system results seem to support its conceptual superiority and use as a valid public health strategy to reduce obesity and related disorders. This FOP system needs continuous confirmations and extension, not only at a national level but also at an international level, and NutrInform Battery must remain open to further improvements (see below).

## Highlights and recommendations

Overweight and obesity are multi-factorial diseases with a heavy impact on the health and life quality of the population. Within a general approach considering the whole lifestyle, dietary control is probably the most helpful tool to prevent this condition, which is often too complex to be clinically handled due to the limited availability of efficacious interventions. Therefore, the information handed out to the consumer is considered an essential tool to favour proper food choices and prevent overweight. This reasoning goes far beyond obesity and related diseases; proper food education and healthy nutrition prevent the onset of NCDs, improving the population's overall health status and longevity in good psychophysical conditions.

In Europe, within the Community strategy to reduce overweight and obesity, an important debate is ongoing on supplementary front-of-pack labelling systems that should help consumers make healthier and more favourable food choices; the chosen scheme will be adopted in the entire European Community. Nutri-Score is the supplementary nutritional labelling system proposed by French scientists. It classifies foods into five categories: A, dark green (the best) to E, red (the worst) based on applying a specific algorithm. It is an interpretative/evaluation and non-informative system that does not provide any information on the nutritional characteristics of the food (composition, content in nutrients, and others). Contrary to what is emerging from most recent studies, the importance (and hence the weight) of ingredients with “undesirable” effects in Nutri-Score, is much greater than that of ingredients with beneficial effects. Moreover, Nutri-Score is based on the composition of 100 g of foods, regardless of the portion sizes, which are essential to monitor the energy intake properly and control the weight. The underlying nutritional approach is indiscriminate since it should be proposed to the entire population without considering the distinct nutritional needs and energy intake of subjects with specific health issues (e.g., overweight and obesity, hypertension, dyslipidemia).

Studies performed and published using Nutri-Score do not provide convincing evidence to support the hypothesis that adopting this FOP information system facilitates the maintenance of a proper BMI or reduces the probability of developing overweight or obesity. The system developed and proposed by Italy (i.e., NutrInform Battery) looks more flexible and potentially more informative. FOP is one of the elements of a broader framework of a training process that makes the consumer aware. The FOP cannot be used alone, and it is necessary to give the consumer the key to reading it in a broader and more articulated context than healthy food choices. In the future, to improve the NutrInform Battery system, the FOP could be implemented with the results from the application of the Nutrient and Hazard Analysis of Critical Control Point (NACCP) [38], which allows to evaluate and monitor the presence and quantity of a specific nutrient along the entire supply chain. The last step involves testing the effect on the consumer. This process has already been approved in various ministerial documents in Italy.

## Strength and limits

We are aware of the limitations and biases of position paper like the present one: (1) it is more likely to include only research selected by the authors; (2) it fails to content-code the studies either for theoretically important aspects or for aspects that gauge methodological quality—the result is that often the accuracy of the paper's claims about the characteristics of the studies and the quality of their methods is difficult to judge; (3) it rarely employs peer-reviewed methodologies, duplicates the curation of evidence, and often fails to disclose study inclusion criteria; (4) it may unrecognise the procedures used to reach and offer conclusions about the nature of quantitative or qualitative empirical literature. The strength of the present position paper is to have tried to overcome these significant limits and offer the experts’ opinion of the current knowledge on this complex, multifaced topic. We hope to have reached an adequate level of accuracy on a lifestyle topic highly relevant to the general population.

## What is already known on this subject?

Various FOP nutritional labels have been realised to enhance consumers' knowledge of nutritional food quality and promote healthier decisions. However, few studies have examined the consequences of FOPs on consumers' subjective knowledge and learning towards nutritional values and correctness of foods to prevent the development and combat diffusion of obesity in the different cultural systems and countries.

## What does this study add?

In the present position paper, we compare two FOP nutritional labels, the NutrInform Battery and Nutri-Score system, suggesting that the first can help consumers better than the second system to understand nutritional information, potentially improving dietary choices. In particular, our paper underlines that the published results on the Nutri-Score system do not provide convincing evidence to support the hypothesis that adopting this FOP information system facilitates the maintenance of a proper BMI or reduces the probability of developing overweight or obesity, despite its wide adoption by various countries and public institutions. On the contrary, the NutrInform Battery system looks more flexible and potentially more informative in this context of public health.

## Footnotes

1 Italian Association of Dietetics and Clinical Nutrition (ADI) Foundation, Italian Obesity Network (IO-NET), Italian Society for the Study of Eating Disorders (SISDCA), Italian Society of Alimentary Sciences (SISA), Italian Society of Paediatric Endocrinology and Diabetology (SIEDP), Italian Association of Dietetics and Clinical Nutrition (ADI), Italian Society of Pediatric Nutrition (SINUPE), Italian Obesity Society (SIO).
